# The Enhanced Whey Protein Oral Bioavailability and Muscle Anabolism Ability by a Simple and Effective Piperine‐Whey Protein Synergistic Codelivery System

**DOI:** 10.1002/advs.202522455

**Published:** 2026-03-26

**Authors:** Qing Yue, Kaiwen Wu, Bing Wei, Guiling Song, Congyu Sun, Xing Li, Xiangyu Liu, Zhaoxiang Ma, Mingchun Lv, Jasper Landman, Yuan Li

**Affiliations:** ^1^ Research Center of Food Colloids and Delivery of Functionality College of Food Science and Nutritional Engineering China Agricultural University Beijing China; ^2^ Beijing Competitor Sports Science & Tech. Co., Ltd. Beijing China; ^3^ Frontier Technology Research Institute of China Agricultural University in Shenzhen Shenzhen China; ^4^ Physics and Physical Chemistry of Foods Wageningen University and Research Wageningen Netherlands

**Keywords:** nanoemulsion, piperine, sarcopenia, skeletal muscle, whey protein

## Abstract

The strength of skeletal muscle is critical for daily function and exercise capacity. Whey protein (WP) is widely utilized to support muscle strength, but WP alone is insufficient to achieve desired muscle synthesis. Here, a simple and effective piperine (Pip)‐WP synergistic supplementation system was developed. A nanoemulsion with high interfacial‐volume WP layer, designated as WP(Pip), was prepared with 10 wt.% WP and Pip loaded in the oil phase. Compared with the single dose of WP and Pip, the co‐delivered system of WP(Pip) combined with major WP can increase in vivo WP and Pip absorption, with increase of 6.10% and 890%, respectively. The mechanism of Pip for WP hydrolysate absorption is investigated in a Caco‐2 monolayer model. Pip enhances WPH bioavailability by reversibly modulating epithelial tight junctions. Increased circulating free amino acids, together with Pip, synergistically activate mTOR signaling pathway in skeletal muscle through direct and indirect regulation. This promotes efficient utilization of absorbed WP for muscle protein synthesis and improves exercise performance. Meanwhile, the WP(Pip) co‐supplementary system mitigates muscle fatigue and damage in healthy mice and in a dexamethasone‐induced sarcopenia model, respectively. These findings suggest a strategy to enhance protein absorption and translate dietary protein intake into muscle‐building outcomes, with potential nutritional and clinical relevance.

## Graphical abstract

WP(Pip) is a mixture of whey protein and piperine‐loaded nanoemulsion stabilized by WP through microfluidic homogenization. Acting as a “smart key,” piperine opens epithelial tight junctions to enhance absorption of whey protein and, thereby increasing circulating amino acid availability, synergistically up‐regulates the mTOR signaling pathway in skeletal muscle, thereby promoting the efficient utilization of absorbed WP in muscle protein synthesis. WP(Pip) showed beneficial effects in both acute exercise and sarcopenia models.

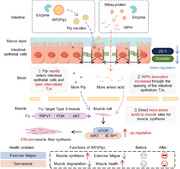

## 1 Introduction

Skeletal muscle is the most abundant tissue in the human body, accounting for nearly 40% of adult body mass, and its integrity is essential for maintaining physical performance and overall physiological function [1]. Acute exercise depletes amino acids and causes muscle damage, while sarcopenic populations exhibit pronounced anabolic resistance [2]. Consequently, higher protein intake and bioavailability beyond habitual diets are required to effectively support muscle growth. Whey protein (WP), a byproduct recovered from whey during cheese processing, is rich in essential amino acids and branched‐chain amino acids, and its composition closely resembles that of skeletal muscle proteins, thereby promoting muscle protein synthesis [3, 4]. Although WP exhibits relatively high digestibility and absorption among commercial protein supplements, it remains insufficient to meet the protein requirements of specific populations [5]. Increasing intake can partially compensate for limited absorption. However, this strategy imposes a metabolic burden and may lead to adverse health problems [6]. Muscle protein synthesis is primarily regulated through the mTOR signaling pathway, in which amino acids play a key role [7]. Accordingly, appropriate supplementation with exogenous proteins holds promise for promoting muscle anabolism. However, the simple and effective approaches for further enhancing protein utilization, particularly in a muscle‐targeted manner, remain limited.

Piperine (Pip) is an alkaloid derived from black pepper that has been shown to enhance the absorption of hydrophobic small molecules such as curcumin by inhibiting metabolic enzymes and efflux transporters [8, 9]. Previous *ex vivo* studies have reported that Pip can enhance amino acid uptake [10]. However, further evidence is lacking. Other studies have reported that Pip can interfere with the myosin state, thereby perturbing the balance between muscle protein synthesis and degradation [11]. Meanwhile, reports detailing how Pip enhances amino acid absorption and subsequently promotes muscle protein synthesis remain scarce. It should also be noted that Pip is highly hydrophobic (LogP = 3.5), prone to aggregation and environmentally labile, posing challenges for preserving its bioactivity and ensuring effective delivery to the small intestine [12]. Employing delivery technologies for targeted, controlled release is an effective strategy to enhance small‐molecule absorption and utilization [13, 14]. To enable effective absorption of Pip in the jejunum and ileum, the carrier must withstand gastric acidity and enzymatic degradation during gastric transit and subsequently achieve efficient intestinal release to ensure adequate exposure to and uptake by the epithelial cells [15]. Previous work demonstrated that WP acts as an effective carrier for hydrophobic small molecules [16]. With the design of advanced controlled‐release systems, WP–based carriers can achieve targeted delivery of such compounds [17]. Hydrophobic molecules including curcumin, mangiferin, and raphanin have been encapsulated in WP carriers and delivered to gastric and intestinal segments in a controlled manner [18–20].

In this work, we investigate whether and how co‐supplementation of Pip and WP exhibits a synergistic effect on enhanced protein absorption and muscle protein synthesis. First, the effect of Pip and WP on absorption and muscle synthesis in rat models was evaluated, and then the potential mechanism by which Pip enhances WPH uptake using Caco‐2 monolayers was explored. Next, the combined effect of Pip and WP supplementation on exercise endurance and muscle protein synthesis in mouse models was examined. Finally, the effect of the combined intervention in mitigating chronic muscle loss and oxidative stress in a dexamethasone‐induced sarcopenia mouse model was assessed. These Pip and WP co‐supplementary systems show a high potential in improving protein absorption and enabling targeted utilization in muscle synthesis.

## 2 Results and Discussion

### 2.1 Preparation and Characterization of WP(Pip) Nanoemulsion

**FIGURE 1:**
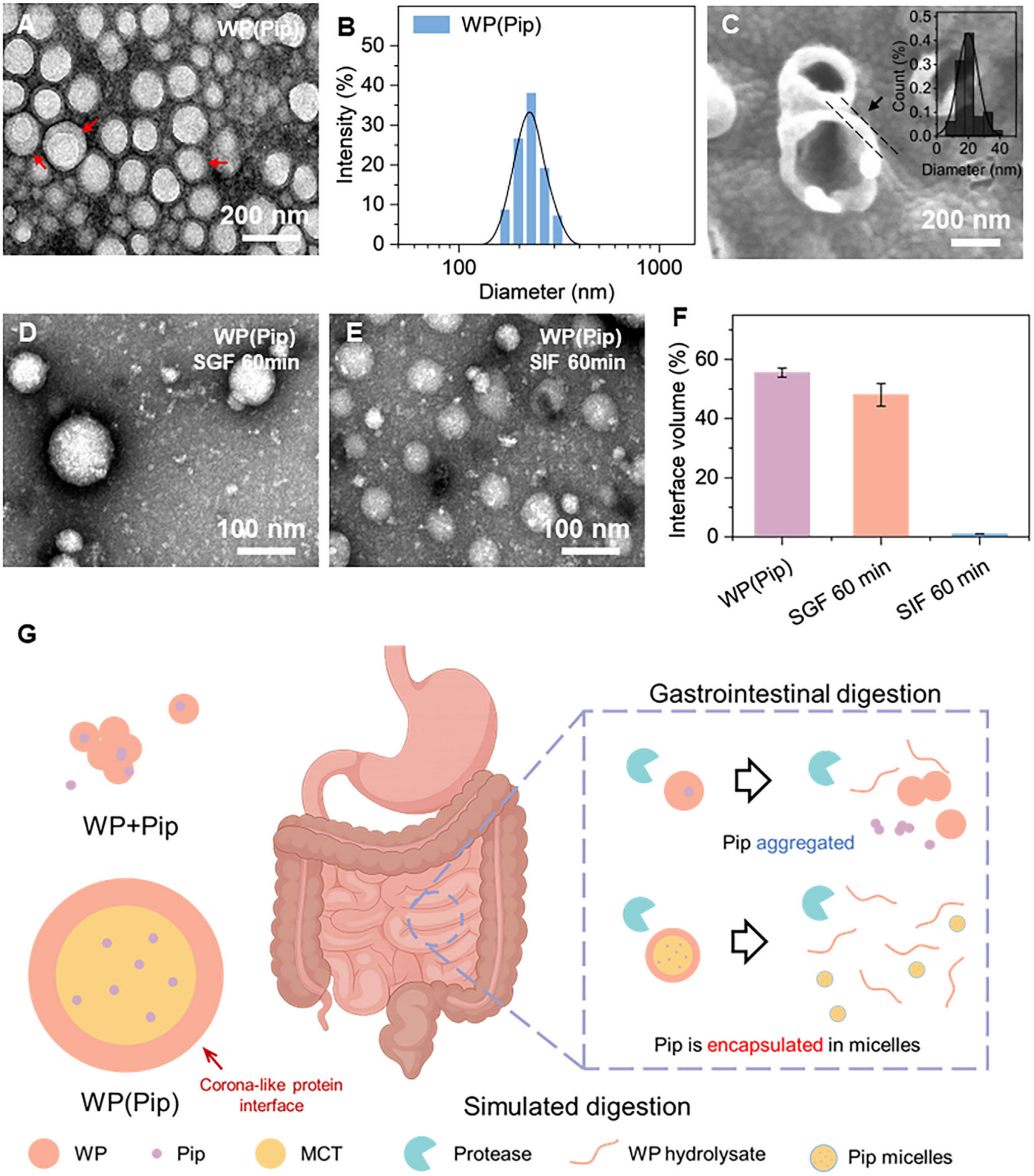
Characterization of WP(Pip) nanoemulsion. (A) TEM image of fresh prepared WP(Pip), The red arrows indicate typical protein “corona”, scale bar = 200 nm. (B) Droplet size of WP(Pip). (C) Cryo‐SEM image and interface thickness of WP(Pip), scale bar = 200 nm. (D) TEM image of WP(Pip) in SGF for 60 min, scale bar = 100 nm. (E) TEM image of WP(Pip) in SIF for 60 min, scale bar = 100 nm. (F) Interface volume of WP(Pip) before simulated digestion, simulated gastric digestion for 60 minutes, and simulated intestinal digestion for 60 minutes. (G) Proposed digestive mechanisms of WP+Pip and WP(Pip), which distinct fates during gastrointestinal digestion.

Nanoemulsification can improve the water dispersibility and oral bioavailability of hydrophobic piperine (Pip). Then Pip oil dispersion was mixed with 10 wt.% whey protein (WP) dispersion by first high‐shear dispersion and then passed twice through a microfluidic homogenizer at 12,000 psi to obtain a stable WP(Pip) nanoemulsion. Since the protein concentration was quite high so only a small portion of WP was adsorbed at the WP(Pip) interface, most of WP may remain free state in the WP(Pip) mixture. Additionally, the formulation of WP+Pip was prepared by simple mixing. As shown in Figure S1, WP appeared as heterogeneous particles with sizes ranging from 20 to 50 nm. When Pip was simply mixed with WP, WP aggregation was observed (Figure S2A). The transmission electron microscope (TEM) and DLS revealed that WP(Pip) nanoemulsion exhibited a 228.3 nm droplet size (Figure 1A,B). Cryo‐SEM was also employed to capture the state of WP(Pip) droplets in water, revealing a shell thickness of 10–32 nm, which also indicates a high interfacial volume fraction (Figure 1C). The interfacial volume was calculated as the subtraction between the droplet and oil‐phase volumes, revealing that these nanoemulsion droplets possessed a high interfacial volume fraction of nearly 56% due to a high protein concentration and two passes of homogenization used. These findings confirm that WP forms a thick protein layer at the interface in a manner resembling a protein “Corona”. Interfacial tension measurements demonstrated that a 10 wt.% WP concentration leads to a fast attainment of equilibrium (Figure S3). microfluidic homogenization disrupted the original coarse droplets and the proteins adsorbed at the interface, resulting in the reformation of uniform small droplets [21]. Owing to the ability of high protein concentration to quickly stabilize the interface, proteins were promptly redistributed onto the surface of small droplets, so that kinetically stable nanoemulsion could form quickly.

The delivery of bioactive compounds to target location is essential for exerting their biological effects [22]. WP is mainly absorbed in the small intestine while Pip is reported to open intestinal epithelial tight junctions (TJs). WP+Pip simple mixture still kept aggregation during both simulated gastric and intestinal digestion (Figure S2B,C). In contrast, WP(Pip) nanoemulsion showed a reduced droplet size and slightly decreased interfacial thickness during gastric digestion, followed by further droplet shrinkage and nearly undetectable interfacial layers during intestinal digestion (Figure 1D–F). Consistent with this observation, WP remained stable in the gastric phase but was quickly digested in the intestine (Figure S4), suggesting that the WP interfacial layer of droplets during intestinal digestion may be replaced and stabilized by WP hydrolysates and bile salts. Meanwhile, microfluidic homogenization and emulsification might affect the gastric digestion slightly, which may ultimately influence WP absorption ratio [23]. As described in Figure 1G, we speculate that WP(Pip) forms smaller Pip‐loaded droplets during the digestion stage in vivo, and promotes translocation across the intestinal barrier, thereby enhancing the absorption of Pip dispersed in MCT [24]. Moreover, differences in the dispersion state of Pip between WP+Pip and WP(Pip) may also lead to differences in Pip bioaccessibility. This will be investigated in the following section.

### 2.2 Pip enhances WP Protein Bioavailability and Muscle Protein Synthesis In Vivo

**FIGURE 2:**
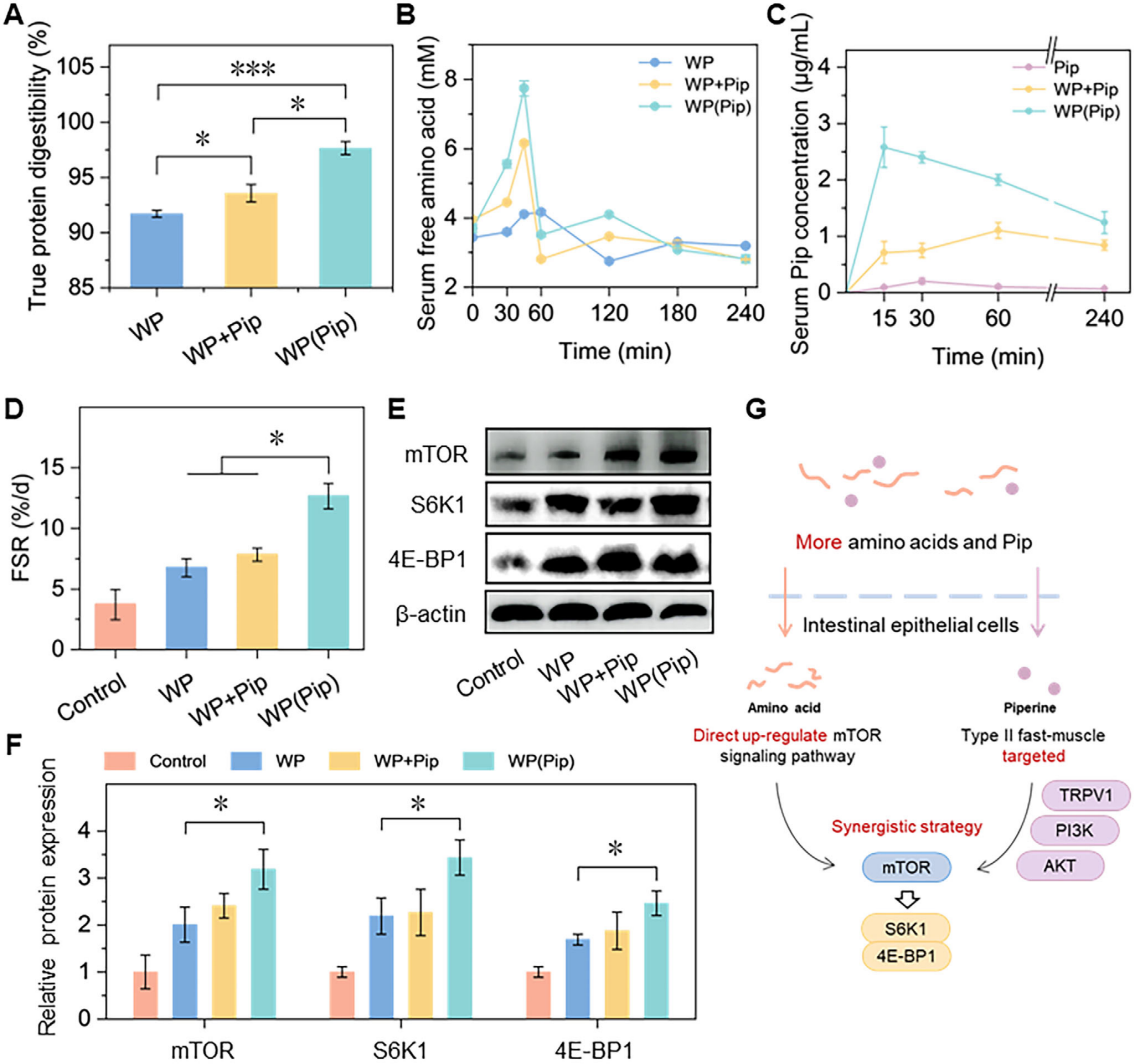
WP(Pip) enhances WP and Pip absorption and promotes muscle protein synthesis. (A) True protein digestibility of WP, WP+Pip, and WP(Pip) groups over 12 h. (B) Plasma free amino acids concentrations at different time points after oral administration of WP, WP+Pip and WP(Pip). (C) Plasma Pip concentrations at different time points after oral administration of Pip, WP+Pip and WP(Pip). (D) FSR of skeletal muscle protein in the Control, WP, WP+Pip, and WP(Pip) groups. (E) Representative western blots analysis of mTOR, S6K1, and 4E‐BP1 in skeletal muscle 1 h after oral administration of WP, WP+Pip, and WP(Pip). (F) Quantification of western blot results in (E). (G) Proposed mechanism by which WP(Pip) promotes muscle protein synthesis. Data are presented as mean ± SD (n = 3). Statistical significance was assessed by Student's t‐test. *p ≤ 0.05, **p ≤ 0.01, ***p ≤ 0.001.

Accurate evaluation of protein utilization in complex physiological conditions relies on in vivo absorption studies [25]. The true protein digestibility of protein was measured by calculating the nitrogen content in feces after oral administration reflecting the protein utilization after digestion. Then the true protein digestibility of various WP formulations was measured in SD rats. Surprisingly, the supplemental Pip significantly increased true protein digestibility, WP+Pip and WP(Pip) reaching 93.58% and 97.66%, respectively, corresponding to increases of 2.04% and 6.10% relative to the only WP group (Figure 2A). This suggests that Pip may be one of the key factors promoting WP absorption. The bioavailability of WP after oral uptake with different WP formulations was further measured by the blood amino acid level. The WP(Pip) group exhibited a 1.86‐fold higher blood free amino acid concentration at *T_max_* than the WP group, with the peak time advanced to 45 min and levels returning to baseline at 60 min (Figure 2B). Compared with previous reports suggesting that the degree of hydrolysis has a limited contribution to the after a meal amino acid appearance rate of whey protein, Pip appears to further enhance the bioavailability of WP [26, 27]. Moreover, both WP+Pip and WP(Pip) markedly enhanced the bioavailability of Pip after oral uptake of different formulations. Notably, WP(Pip) accelerated *T_max_* of Pip to 15 min (Figure 2C and Table S1). This may be attributed to rapid gastric emptying associated with the low viscosity of WP(Pip) (Figure S5) and to potential changes in protein structure induced by microfluidic homogenization, both of which may promote absorption of WP. Subsequently, bile salts convert the emulsion into smaller micelles, thereby promoting the quick translocation of Pip across the intestinal mucosa. [28] Clearly, the co‐administration of WP and Pip exhibited a synergistic effect, further promoting the bioavailability of both ingredients in vivo.

An increased protein intake does not necessarily imply that the additional amino acids are fully used for muscle protein synthesis. Therefore, it is necessary to determine whether oral treated WP+Pip and WP(Pip) with equal WP concentration enhance muscle anabolism by promoting plasma free amino acid concentrations. Exploiting the incorporation of free amino acids into muscle protein, L‐phenylalanine (Ring‐D5) was employed as an amino acid tracer to assess the contribution of blood free amino acids to muscle protein synthesis. Protein‐bound L‐phenylalanine (Ring‐D5) in hydrolyzed gastrocnemius muscle was then quantified to calculate the fractional synthesis rate (FSR). WP supplementation increased the FSR across all groups (Figure 2D). Notably, the WP(Pip) group exhibited the highest FSR, which was significantly higher than that observed in the WP+Pip group. These results suggest that WP(Pip) and WP+Pip may differ in the mechanisms by which circulating free amino acids are mobilized and used for muscle protein synthesis.

Further study of the mTOR signaling pathway showed that WP(Pip) markedly up‐regulated mTOR and its downstream effectors S6K1 and 4E‐BP1 (Figure 2E, F), indicating that circulating free amino acids under the WP(Pip) condition are being efficiently channeled into skeletal muscle protein synthesis. The increased amino acid up‐regulate the mTOR expression, promoting protein of downstream effectors 4E‐BP1 and S6K1 and thereby driving protein synthesis [29]. Circulated Pip preferentially targets type II fast‐twitch muscles (e.g., the gastrocnemius), shifting myosin heads from the super‐relaxed state into a disordered relaxed state and thereby increasing skeletal muscle metabolic rate [11]. As a known TRPV1 agonist [30], Pip activates TRPV1 to up‐regulate the PI3K/AKT signaling pathway [31], and in the presence of increased circulating free amino acids it further enhances mTOR pathway activation [32]. Conversely, WP+Pip produced only modest up‐regulation of mTOR, S6K1, and 4E‐BP1 (Figure 2E, F). Therefore, the Pip together with high level free amino acids concentration synergistically up‐regulates mTOR, rapidly and efficiently leading absorbed protein through the mTOR signaling pathway into increased gastrocnemius muscle synthesis. Under the ordinary conditions, the increased pool of circulating free amino acids is more likely to be routed toward hepatic metabolism and maintenance of systemic energy homeostasis rather than being preferentially used for gastrocnemius muscle protein synthesis [33]. While, the additional free amino acids in blood may recruit to the muscle synthesis instead of providing energy after taken WP(Pip), reducing the risk of metabolic imbalance of α‐keto acids [34]. Taken together, these findings suggest that Pip may play a key role in enhancing WP absorption and utilization in skeletal muscle. A higher Pip exposure may promote targeted mobilization of circulating free amino acids to promote muscle fibers synthesis through a synergistic strategy through the direct up‐regulate of mTOR by amino acid and an indirect up‐regulate of mTOR by Pip, thereby promoting muscle protein synthesis in those fibers (Figure 2G).

### 2.3 Pip promotes WP Absorption by Opening Paracellular Pathway in Human Colorectal Adenocarcinoma (Caco‐2) Monolayer

**FIGURE 3:**
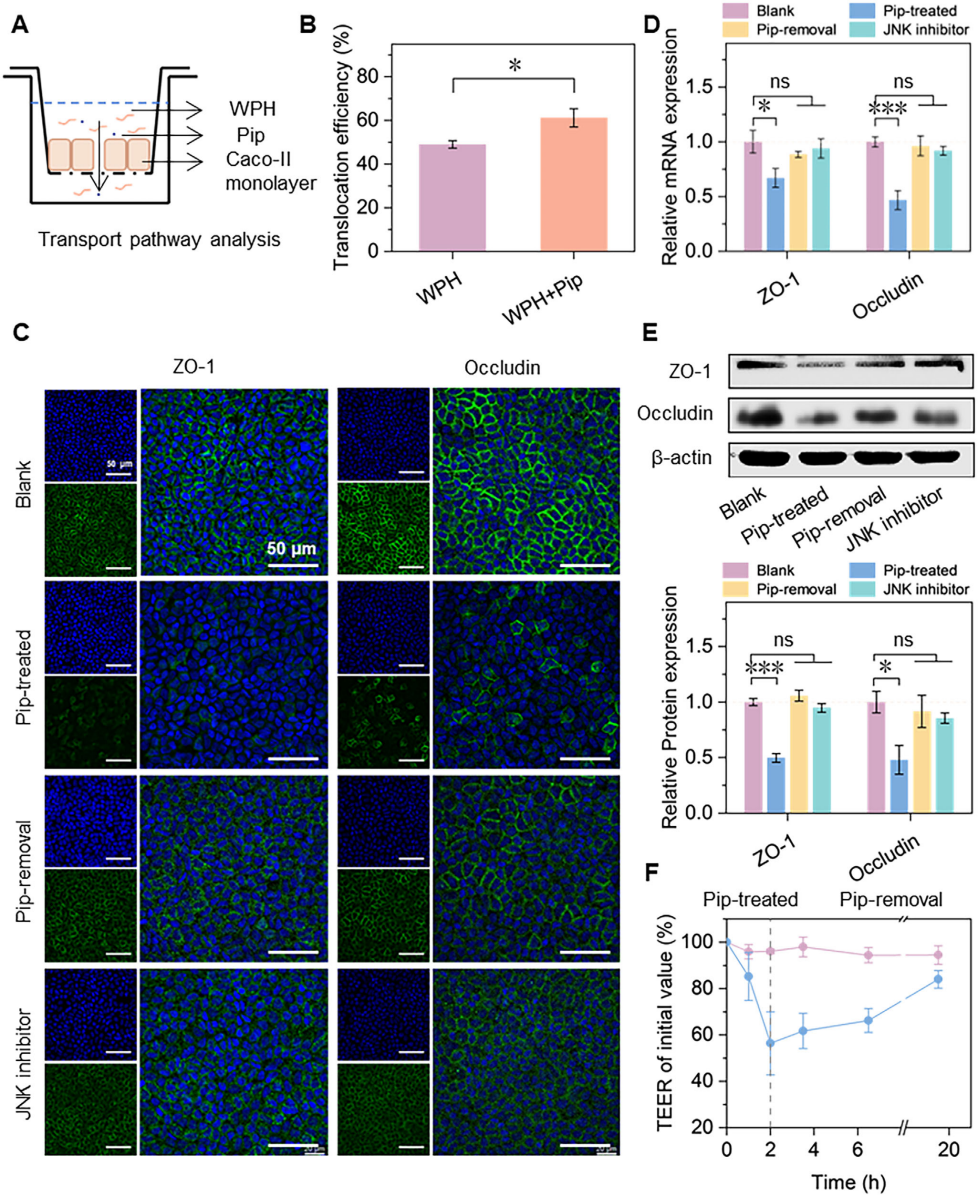
Pip promotes WP hydrolysates (WPH) absorption by modulating tight junctions in Caco‐2 monolayers. (A) Schematic of the Transwell assay used to establish a Caco‐2 monolayer. (B) Permeability of WPH across the Caco‐2 monolayer in the presence or absence of Pip. (C) Representative immunofluorescence images of the TJ proteins ZO‐1 and Occludin under the indicated treatments, the scale bar = 50 µm. Nuclei are stained blue, and ZO‐1 and occludin are stained green. (D) Relative mRNA expression of ZO‐1 and Occludin under the indicated treatments. (E) Relative protein expression of ZO‐1 and Occludin under the indicated treatments. (F) Transepithelial electrical resistance (TEER) of the Caco‐2 monolayer in the presence or absence of Pip. Data are presented as mean ± SD (n = 3). Statistical significance was assessed by Student's t‐test. *p ≤ 0.05, ***p ≤ 0.001.

The above results suggested that Pip may be a key factor contributing to improved WP utilization and enhanced muscle anabolism. However, further evidence is still needed to clarify the specific mechanism by which Pip promotes WP absorption. Given that the small intestine is the primary site of nutrient absorption and WP remains relatively stable in the stomach but is quickly hydrolyzed during intestinal digestion (Figure S4), WP hydrolysates (WPH) was prepared by incubating WP in simulated gastric fluid (SGF) for 2 h followed by simulated intestinal fluid (SIF) for 2 h, which represents the major form available for intestinal absorption. To clarify the molecular mechanism underlying Pip‐enhanced WPH transport, a Caco‐2 monolayer model was established (Figure 3A). The addition of Pip markedly increased translocation efficiency of WPH across the Caco‐2 monolayer (Figure 3B). Concurrently, immunofluorescent signals of the TJ proteins ZO‐1 and Occludin were substantially attenuated after Pip treatment, indicating compromised TJs integrity (Figure 3C). The changes in relative mRNA and protein expression were consistent with these observations, revealing significant down‐regulation of ZO‐1 and Occludin (Figure 3D,E). This indicates that Pip may promote WPH absorption by down‐regulating TJs and increasing epithelial permeability. Irreversible disruption of intestinal epithelial TJs can permit translocation of harmful metabolites and thereby increase the risk of inflammation [35]. Following a 2‐hour exposure to Pip and subsequent removal, transepithelial electrical resistance (TEER) progressively increased over the ensuing 18 hours, indicating reversible TJ opening and recovery. Concurrently, immunofluorescent signals, relative mRNA and protein levels of the TJs proteins Occludin and ZO‐1 were also restored (Figure 3C–E). These findings indicate that Pip‐induced increases in epithelial permeability are reversible and biocompatible.

To determine the molecular pathway by which Pip modulates TJs in the Caco‐2 monolayer, the monolayers were pretreated with a JNK‐specific inhibitor to Pip exposure. Following JNK inhibition, Pip treatment did not produce significant changes in ZO‐1 and Occludin immunofluorescence intensity, relative mRNA expression or protein levels compared with the blank group (Figure 3C–F). These findings indicate that JNK activity is required for the Pip‐mediated regulation of TJs and are consistent with a mechanism in which Pip promotes WPH translocation by activating JNK to modulate ZO‐1 and Occludin and reversibly increase epithelial permeability.

To improve the bioavailability of WP, extensive studies have been conducted to enhance its digestibility and utilization efficiency. Common approaches mainly focus on heat treatment, mechanical processing, and pre‐enzymatic hydrolysis [23]. Heat treatment and mechanical processing can alter protein conformation, making WP more susceptible to proteolytic hydrolysis during digestion and thereby potentially improving its utilization [36]. Pre‐enzymatic hydrolysis generates WPH with lower molecular weight, which may further improve WP utilization during subsequent digestion [37]. However, few reports have shown that external processing approaches can alter the targeted utilization of WP. By contrast, Pip may not only promote WPH absorption by modulating intestinal epithelial TJs, but also promote the targeted utilization of free amino acids in the gastrocnemius muscle, thereby enhancing the targeting of WP toward muscle protein synthesis. Therefore, Pip promotes the targeted utilization of WP in the gastrocnemius muscle through a novel strategy.

### 2.4 WP(Pip) Enhances the Sports Performance and Attenuates Acute Muscle Damage on Exercise Model

**FIGURE 4:**
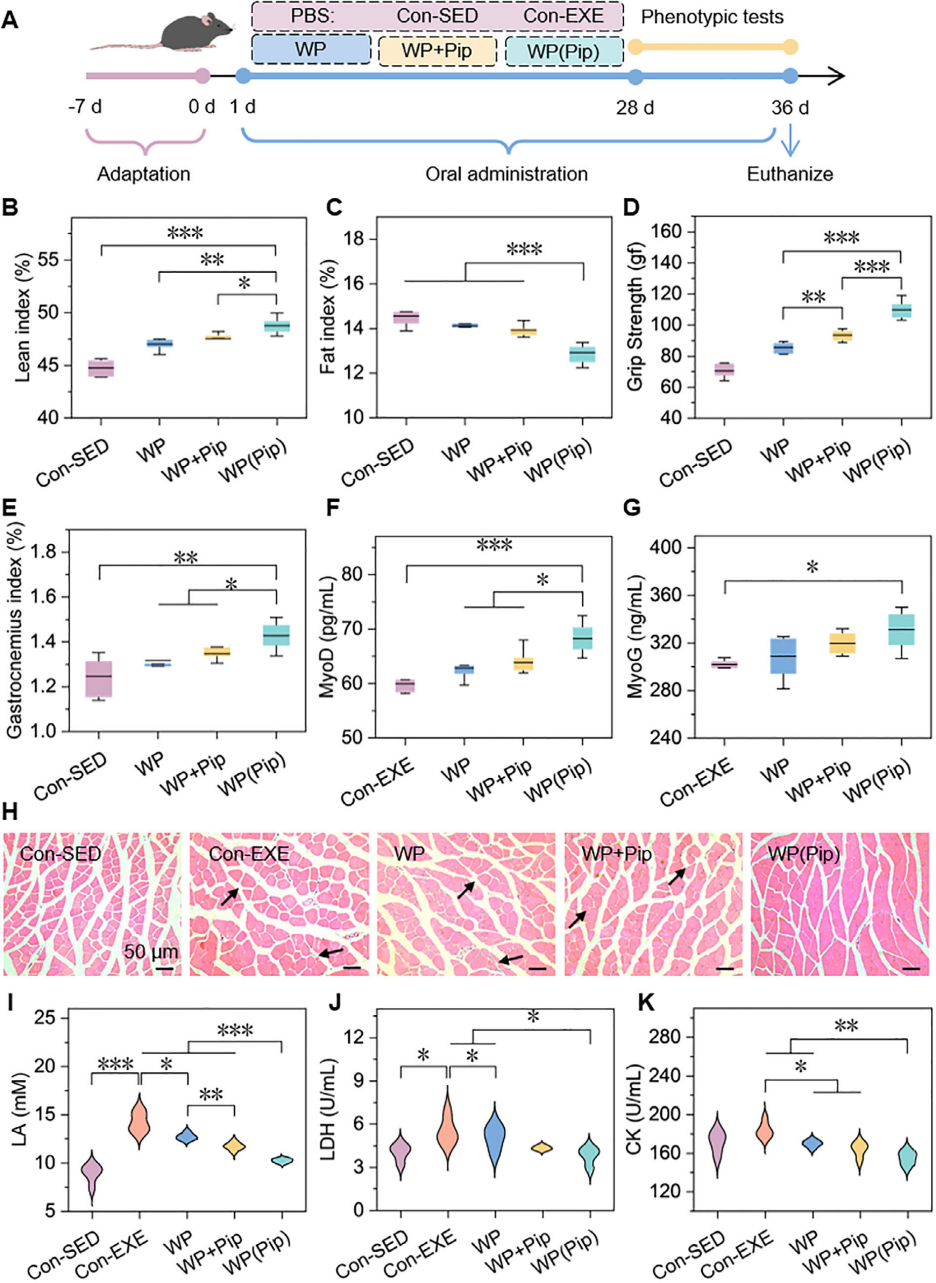
WP(Pip) enhances muscle strength in exercised mice model. (A) Schematic diagram of the experimental design. (B) Lean mass content. (C) Fat mass content. (D) Grip strength. (E) Gastrocnemius muscle index. (F) Serum MyoD levels. (G) Serum MyoG levels. (H) H&E staining of gastrocnemius muscle sections (captured at 10× magnification), The scale bar = 50 µm. (I) Serum lactate levels. (J) Serum LDH levels. (K) Serum CK levels in mice supplemented with PBS, WP, WP+Pip, or WP(Pip) for 36 days. Equivalent daily doses of WP were administered in the WP, WP+Pip, and WP(Pip) groups. Data are presented as mean ± SD (n = 6). Statistical significance was assessed by Student's t‐test. *p ≤ 0.05, **p ≤ 0.01, ***p ≤ 0.001.

High‐intensity anaerobic exercise and resistance training are known to induce microdamage in skeletal muscle fibers, accompanied by lactate accumulation that correlates with exercise‐induced fatigue [38]. Strengthening muscle fiber structure and enhancing functional recovery can mitigate such fatigue. Traditionally, this effect has been achieved via injury‐induced endogenous repair mechanisms triggered by repeated exercise, which subsequently promote fiber remodeling and functional improvement [39]. Given the above findings that Pip enhances WP uptake and promote muscle synthesis in 1 h, we next assessed whether prolonged co‐supplementation could ameliorate acute exercise‐induced muscle damage and fatigue (Figure 4A). The 36‐day intervention did not cause significant increase in serum lipopolysaccharide (LPS) or IL‐1β, suggesting that the Pip‐mediated, reversible opening of epithelial tight junctions did not result in potential intestinal barrier damage or microbial translocation (Figure S6). Although no significant between‐group differences in body weight gain were observed (Figure S7), WP supplementation was associated with increased lean mass and decreased fat mass (Figure 4B,C). Notably, mice receiving Pip displayed a higher lean‐to‐fat mass ratio, which may be attributed to a Pip‐mediated increase in basal metabolic rate [40]. All groups receiving WP supplementation exhibited increased forelimb grip strength (Figure 4D). The gastrocnemius is a major skeletal muscle of the lower limb that governs explosive force and short‐duration strength and is prone to fatigue and microdamage during exercise [41]. The WP(Pip) group exhibited an increased gastrocnemius index with significant up‐regulation of the myogenic transcription factors MyoD and MyoG (Figure 4E–G), which act downstream of S6K1. These findings suggest that WP(Pip) exerted a sustained stimulatory effect on muscle protein synthesis in healthy mice, potentially by promoting both myogenic differentiation and fiber maturation. Muscle accretion generally contributes to improved exercise performance.

In the swim‐to‐exhaustion test, mice supplemented with WP(Pip) exhibited the longest swim times, indicating enhanced endurance and reduced fatigue (Figure S8). Given the differences in gastrocnemius index, we examined muscle histology and serum markers after a standardized exercise. The control group displayed pronounced histopathological alterations in the gastrocnemius Con‐SED (control group remained at rest, without a standardized exercise) and Con‐EXE (control group with a standardized exercise), including myocyte shrinkage, disrupted myofibrillar striations, and widened inter‐fiber spaces (Figure 4H). Notably, post‐exercise gastrocnemius sections from the WP(Pip) group exhibited regularly arranged fibers with no obvious inter‐fiber gaps. The observed tissue densification is indicative of increased fiber density or hypertrophy. These findings suggest that, even in the absence of high PI3K/AKT/mTOR activation driven by intense exercise, a long‐time WP(Pip) supplement could promote muscle fiber synthesis and differentiation [42]. High‐intensity anaerobic exercise can induce acute myofibrillar damage and increase sarcolemma permeability, resulting in efflux of intracellular metabolites into the circulation [43]. Compared with the Con‐SED, the Con‐EXE group exhibited significant post‐exercise increases in blood lactate, LDH, and CK activities (Figure 4I–K). 36‐day supplementation with WP(Pip) significantly attenuated these exercise‐induced marker elevations, suggesting that enhanced muscle mechanical properties and the consequent reduction in relative exercise load may mitigate metabolite leakage following intense exercise. Efflux of intracellular metabolites can contribute to systemic increases in ROS [44]. Accordingly, blood MDA was assessed as a marker of lipid peroxidation, and activities of endogenous antioxidant enzymes (SOD and GSH‐px) were measured (Figure S9). As TRPV1 agonist, Pip at a low concentration may involve the Nrf2 signaling pathway. Accordingly, mice supplemented with WP(Pip) exhibited the lowest post‐exercise MDA accumulation and the highest antioxidant enzyme activities [45]. These findings suggest a role for enhanced Pip bioavailability in mitigating systemic oxidative stress following acute anaerobic exercise. In summary, this study demonstrates that a 36‐day regimen of prolonged WP(Pip) supplementation may, via long‐lasting up‐regulate of mTOR signaling pathway, promote local enrichment of free amino acids in the gastrocnemius to enhance muscle growth, thereby attenuating acute exercise‐induced muscle damage and improving exercise performance.

### 2.5 WP(Pip) Attenuates Chronic Muscle Loss and Oxidative Stress on Sarcopenia Model

**FIGURE 5:**
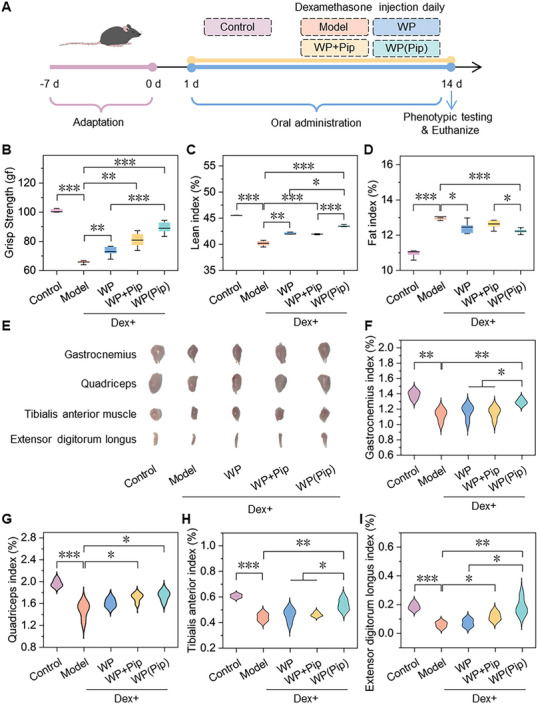
WP(Pip) attenuates Dex‐induced muscle atrophy. (A) Schematic of the experimental design. (B) Forelimb grip strength. (C) Lean mass. (D) Fat mass. (E) Representative images of four skeletal muscles: gastrocnemius, quadriceps, tibialis anterior, and extensor digitorum longus. (F) Gastrocnemius index. (G) Quadriceps index. (H) Tibialis anterior index. (I) Extensor digitorum longus index in mice supplemented with PBS, WP, WP+Pip, or WP(Pip) for 14 days. Equivalent daily doses of WP were administered in the WP, WP+Pip, and WP(Pip) groups. Data are presented as mean ± SD (n = 5). Statistical significance was assessed by Student's t‐test. *p ≤ 0.05, **p ≤ 0.01, ***p ≤ 0.001.

Sarcopenia is a syndrome characterized by the accelerated loss of skeletal muscle mass and function due to physiological or pharmacological factors. Hallmarks of the condition include reduced rates of muscle protein synthesis and chronic low‐grade inflammation. Sarcopenia has occurred as a major public health challenge within global population aging [46]. Affected individuals often present with systemic metabolic dysregulation or impaired immune function, and therefore cannot be reliably rescued from muscle loss by merely increasing dietary protein intake [47]. Dexamethasone (Dex) is a widely used glucocorticoid to induce sarcopenia mice model. Chronic or excessive activation of glucocorticoid receptor pathway by Dex promotes proteolysis and suppresses protein synthesis, finally leading to myocyte apoptosis and inflammation which is typical for sarcopenia. Following 14 days of Dex injections (Figure 5A), Dex‐treated mice exhibited body weight loss (Figure S10), reduced forelimb grip strength (Figure 5B), and a significant decrease in the lean mass‐to‐fat mass ratio (Figure 5C,D), indicating successful induction of the sarcopenic phenotype. WP supplementation partially ameliorated these phenotypic changes across treated groups. Dex‐induced skeletal muscle loss was further evidenced by significant reductions in indices of four key hindlimb muscles (Figure 5E–I). Clinically, protein supplementation is commonly used as an adjunct to exercise interventions, but there is limited evidence that protein supplementation alone reliably reverses sarcopenia [47]. By contrast, WP(Pip) markedly improved Dex‐induced reductions in the mass of four key hindlimb muscles, suggesting that WP(Pip) may mitigate Dex‐driven loss of muscle protein by increasing circulating levels of amino acids and Pip. The previous results already showed that WP(Pip) can promote muscle synthesis (Figure 2D), WP(Pip) may counteract Dex‐induced muscle loss and dysregulated synthesis by maintaining the balance between muscle protein synthesis and degradation.

**FIGURE 6:**
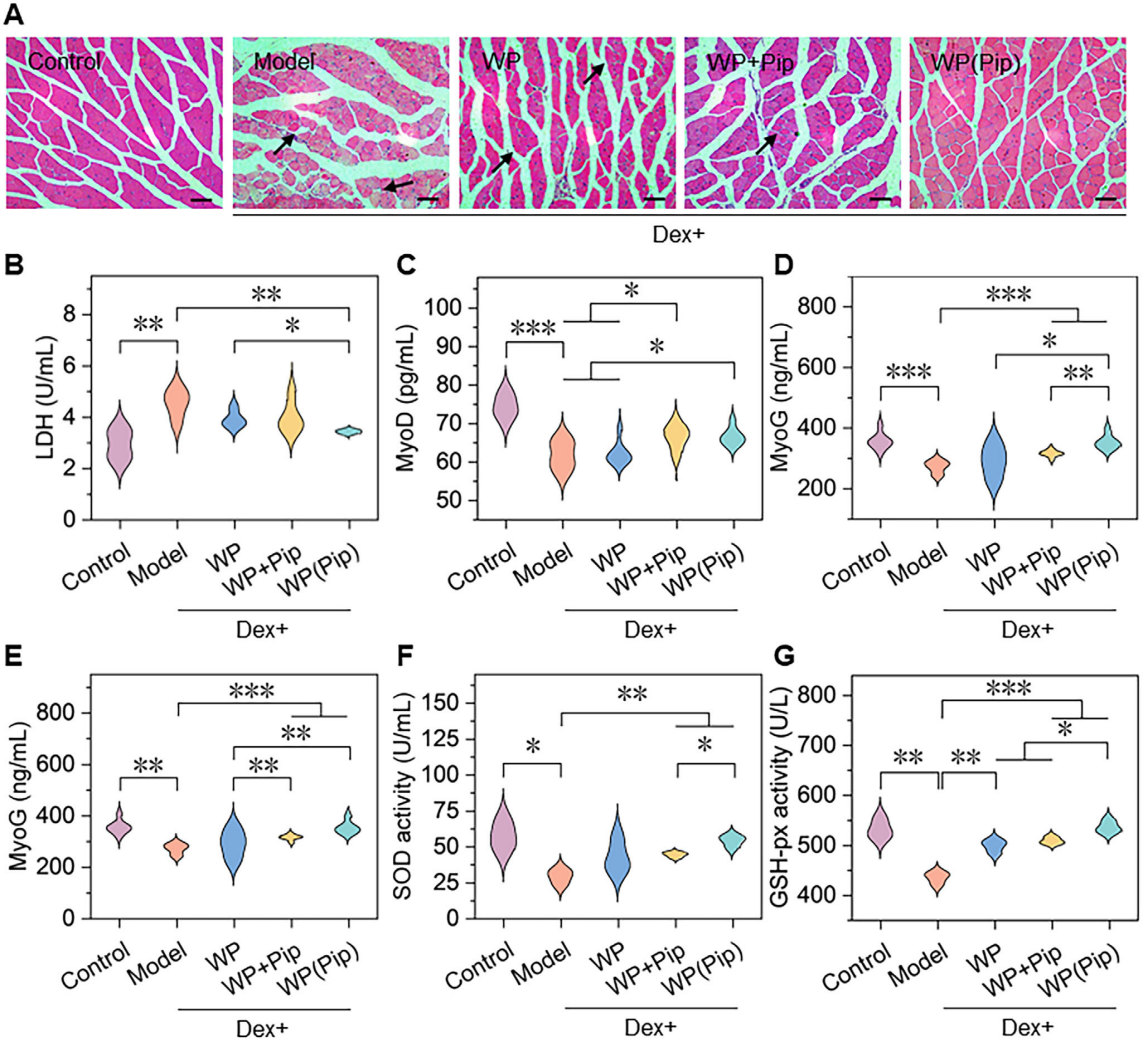
WP(Pip) attenuates Dex‐induced muscle damage and chronic oxidative stress. (A) Representative H&E‐stained sections of the gastrocnemius, the scale bar = 50 µm. (B) Serum LDH levels. (C) Relative expression of MyoD. (D) Relative expression of MyoG. (E) Serum MDA content. (F) SOD activity. (G) Serum GSH‐px activity in mice supplemented with PBS, WP, WP+Pip, or WP(Pip) for 14 days. Equivalent daily doses of WP were administered in the WP, WP+Pip, and WP(Pip) groups. Data are presented as mean ± SD (n = 5). Statistical significance was assessed by Student's t‐test. *p ≤ 0.05, **p ≤ 0.01, ***p ≤ 0.001.

Histological examination of H&E‐stained gastrocnemius after various treatment revealed that Dex treatment induced an atrophy of previously well‐organized fibers, fiber irregularity, and pronounced widening of inter‐fiber spaces (Figure 6A). WP supplementation partially ameliorated these pathological alterations. Notably, WP(Pip) markedly attenuated Dex‐induced myocyte apoptosis. The gastrocnemius sections from WP(Pip) ‐oral treated animals displayed regularly aligned, densely packed fibers with evidence of hypertrophic changes. Consistent with reduced tissue damage, WP(Pip) also suppressed the accumulation of LDH in serum (Figure 6B), supporting its protective effect against skeletal muscle injury. Changes in MyoD and MyoG further substantiate the critical role of WP(Pip) in counteracting Dex‐induced myocyte apoptosis (Figure 6C,D). Mechanistically, Dex acts via the glucocorticoid receptor to upregulate ubiquitin ligases MuRF1 and Atrogin‐1, thereby promoting myosin degradation, while concurrently suppressing the PI3K/AKT/mTOR signaling axis to inhibit protein synthesis [48]. These combined effects are reflected by down‐regulation of MyoD and MyoG (Figure 6C, D). These results are similar as the histological findings, indicating that the protective effect of WP(Pip) involves both attenuation of muscle protein degradation and maintenance or restoration of myogenic transcriptional programs. Supplementation with WP(Pip) significantly attenuated the down‐regulation of MyoD and MyoG by preserving mTOR pathway homeostasis. Notably, MyoG levels in the WP(Pip) group were comparable to those in the Control group, suggesting that WP(Pip) promotes myofiber formation and remodeling, which may act as a key mechanism for counteracting Dex‐induced chronic muscle injury.

Dex acts through the glucocorticoid receptor to suppress mitochondrial biogenesis, leading to chronic oxidative stress. Consistent with this mechanism, serum MDA accumulates while activities of SOD and GSH‐px decline (Figure 6E–G). Endogenous antioxidant defenses and compensatory responses are insufficient to resolve Dex‐induced ROS accumulation, which may predispose to dysregulated inflammation [49]. Supplementation with WP(Pip) attenuated MDA accumulation and rescued antioxidant enzyme activities, suggesting that WP(Pip) treatment preserved muscle mass under chronic insult and also contributed to reducing systemic oxidative stress. However, the precise mechanism by which WP(Pip) reverses Dex‐induced sarcopenia still requires further metabolic validation.

**FIGURE 7:**
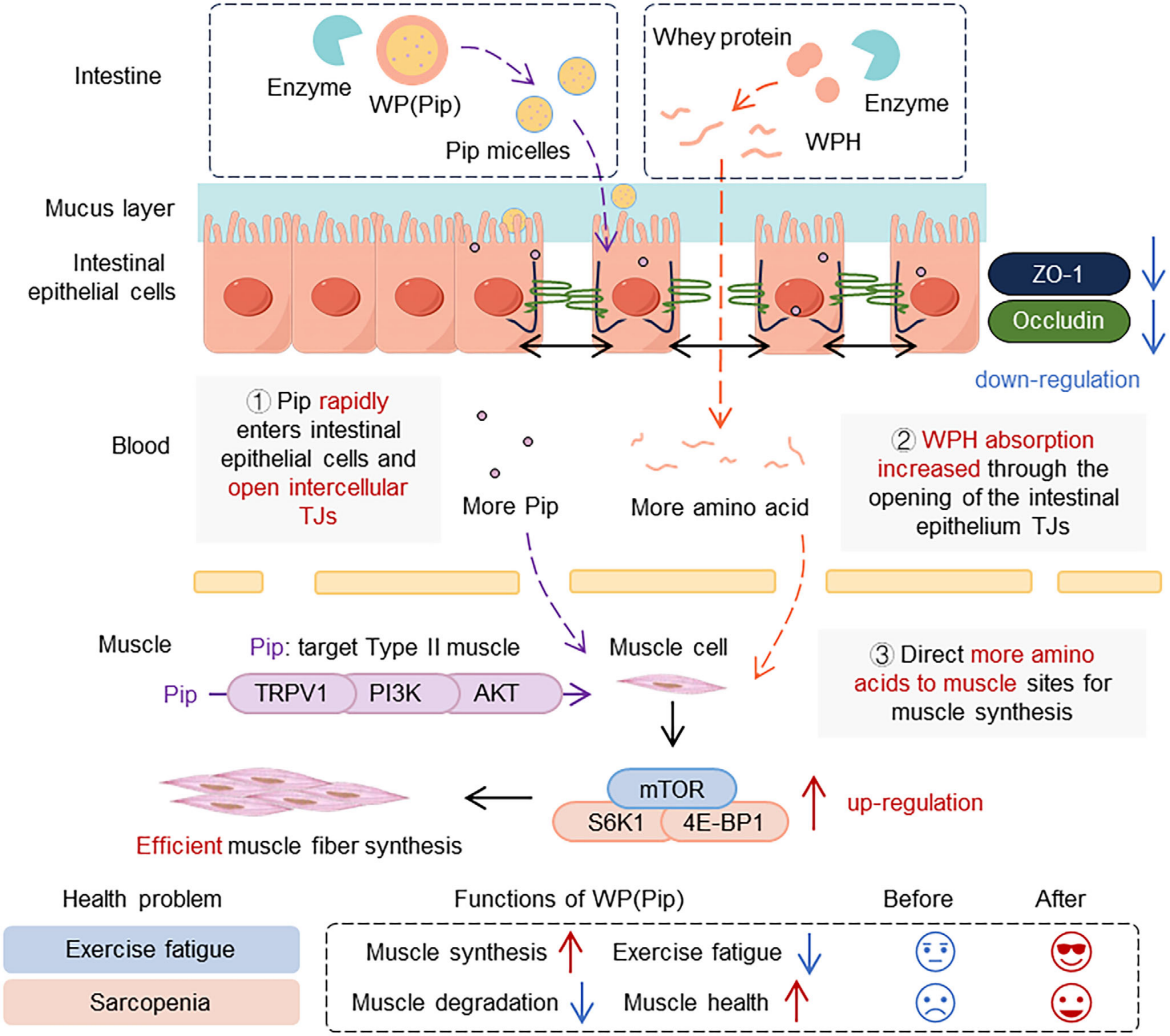
Schematic of the proposed mechanism by which WP(Pip), via the combined action of Pip and WP in nanoemulsion, promotes muscle protein synthesis.

In summary, this study reveals the roles of Pip and WP co‐delivery in muscle anabolism (Figure 7). Under equivalent WP intake, Pip functions as a “master key” that transiently increases paracellular intestinal permeability via JNK‐mediated, reversible down‐regulation of tight‐junction proteins ZO‐1 and Occludin, thereby enhancing the oral bioavailability of both WP and Pip. Modulation of Pip delivery by WP‐based nanoemulsion further elevates systemic Pip exposure. Pip directs circulating free amino acids to type II fast‐twitch muscle. The recruited free amino acids directly activate the mTOR signaling pathway, while Pip may indirectly enhance mTOR activation through the TRPV1‐PI3K‐AKT pathway, thereby facilitating the efficient conversion of absorbed WP into muscle protein. In healthy mice, WP(Pip) co‐supplementary strategy strengthened muscle fiber, improved endurance, and reduced acute exercise‐induced fatigue and injury. In the Dex‐induced sarcopenia model mice, WP(Pip) counteracts muscle fiber loss and ameliorates associated oxidative stress by preserving the balance between muscle protein synthesis and degradation. The novel aspect of this concept is utilizing synergistic effects to strengthen muscle synthesis mechanisms, thereby increasing the absorption and muscle synthesis utilization efficiency of dietary protein and improving exercise‐related functional outcomes.

## 3 Conclusions

In this work, we revealed the mechanism by which Pip promotes the oral bioavailability of WP by reversibly opening the intestinal epithelial TJs. In addition, the nanoemulsified WP(Pip) mixture further improved the bioavailability of both piperine and WP and, under specific conditions, synergistically activated mTOR signaling to support muscle protein synthesis. The strategy promoted muscle fiber strengthening and mitigated exercise‐induced fatigue. Furthermore, in a Dex‐induced sarcopenia mouse model, the strategy helped maintain the balance between muscle protein synthesis and degradation. These results support a feasible strategy to promote muscle protein synthesis by increasing protein absorption and directing its utilization in skeletal muscle.

However, considering the differences in digestion and absorption between small animal models (rats and mice) and humans, a limitation of this study is the lack of validation of WP absorption and muscle growth in large animals or humans. Furthermore, inspired by the findings of the current study, which show that Pip reversibly modulates intestinal epithelial TJs to enhance WP absorption and utilization, this research may inspire further exploration of other multi‐nutrients synergistic systems for application in multi‐functional food.

## 4 Experimental Section

### 4.1 Materials

Whey protein (WP, 95%) was purchased from Agropur Dairy Cooperative (Saint‐Hubert, Canada). The piperine (Pip), acetonitrile, trichloroacetic acid, L‐serine, HClO_4_, were bought from Sigma‐Aldrich (St. Louis, MO. U.S.). OPA solution was purchased from Thermo Fisher Scientific (Waltham, MA. U.S.). L‐Phenylalanine (Ring‐D5), 98% was purchased from Cambridge Isotope Laboratories, Inc (Tewksbury, MA. U.S.). Medium chain triglyceride (MCT) was purchased from Shanghai yuanye Bio‐Technology Co., Ltd (Shanghai, China). Caco‐2 cells were purchased from Peking Union Medical College (received in 2023.11, RRID:CVCL_0025, the cell line was contamination free by STR test). Gibco Minimum Essential Media (MEM), Gibco fetal bovine serum (FBS) were purchased from Gibco (NY, USA). Rabbit monoclonal anti‐ZO‐1, rabbit monoclonal anti‐Occludin, rabbit monoclonal anti‐mTOR, rabbit monoclonal anti‐S6K1, rabbit monoclonal anti‐4E‐BP1, and rabbit monoclonal anti‐β‐actin were purchased from Cell Signaling Technology, Inc. (Danvers, MA. U.S.). Immunol Fluorescence Staining Kit with AF488‐Labeled Goat Anti‐Rabbit IgG and normal goat serum were purchased from Beyotime Biotech Inc. (Shanghai, China). All chemicals were of analytical grade unless otherwise specified.

### 4.2 Preparation and Characterization of WP+Pip and WP(Pip)

For WP+Pip, 1.0 g WP was dispersed in 9 mL deionized (DI) water and stirred for 2 h, then stored at 4 °C overnight. Subsequently, 0.91 mg Pip dispersed in 1 mL deionized water was added to the WP dispersion and stirred for 2 h. The resulting simple mixture of Pip and WP was designated WP+Pip. For WP(Pip), 3.0 g WP was dispersed in 27 mL DI water and stirred for 2 h, then stored at 4 °C overnight. 2.73 mg Pip was added to 3 mL MCT, followed by stirring and heating at 70 °C for 10 min. The WP dispersion and the Pip/MCT phase were mixed at a ratio of 9:1 (v/v) and subjected to high‐shear dispersion at 15,000 rpm for 5 min to obtain a coarse emulsion. The coarse emulsion was then homogenized using a Microfluidics high pressure homogenizers (Lab Type: Nano, Noozle Fluid Technology, Shanghai, China) at 12,000 psi for two passes to yield the final nanoemulsion, designated WP(Pip). Therefore, WP(Pip) should be considered a mixed system consisting of free WP and nanoemulsion droplets, rather than a nanoemulsion alone. Freshly obtained samples were stored at 4 °C for no longer than 3 days.

For characterization, particle/droplet sizes of WP, WP+Pip, and WP(Pip) were determined using a ZETASIZER PRO (Malvern Instruments, UK). Emulsion viscosity during the simulated digestion phase was measured by a Rheometer (AR 1500 ex, TA Instruments, USA) on a rheometer at a shear rate of 50 s^−^^1^, with an equilibration time of 18 s and a measurement duration of 300 s. The distribution of protein in WP(Pip) between the interface and the water phase was estimated by quantifying the free WP in the water phase. Briefly, samples were centrifuged at 10000 ×g for 30 min to enrich the cream layer. The cream layer was then carefully removed, and the remaining water phase and pellet were thoroughly resuspended. The WP content in the water phase was determined using a BCA assay.

### 4.3 In Vitro Simulated Digestion

Simulated gastric fluid (SGF) and simulated intestinal fluid (SIF) were prepared as described in Table S2. For digestion experiments, the WP dispersion was each mixed 1:1 (v/v) with SGF and incubated at 37 °C at 120 rpm for 120 min. Samples were removed at specified time during the gastric phase. Upon completion of the gastric phase, the pH of the mixture was adjusted to 7.0 with 6 M NaOH and the samples were mixed 1:1 (v/v) with SIF. Intestinal digestion proceeded at 37 °C at 120 rpm for 120 min, with samples taken at specified time points for analysis.

For in vitro protein digestibility, residual protein following the digestion phase was quantified by TCA precipitation. Aliquots of the simulated digestion mixtures were mixed with an equal volume of 10 v/v% TCA to precipitate proteins. And then, centrifuged to pellet the protein, the pellet was resolubilized in 1 M NaOH. Undigested protein content was determined using a BCA assay according to the manufacturer's instructions, and digestibility was calculated accordingly. To characterize digestion products, simulated digestion samples were mixed with 5x SDS–PAGE loading buffer at a volume ratio of 4:1, boiled for 5 min, and subjected to SDS–PAGE. Gels were stained and imaged to assess changes in protein banding patterns. Fresh WP+Pip and WP(Pip) as well as digestion samples collected after 60 min of gastric and intestinal digestion were prepared for TEM analysis. Samples were examined and imaged by transmission electron microscopy (JEM‐1400, JEOL, Japan). TEM images were analyzed using ImageJ. For each digestion stage, results were obtained from at least three independent replicate samples. For each sample, measurements of 200 droplets (including overall droplet diameter and oil‐phase diameter) were randomly obtained from at least 10 TEM images. Interfacial volume was calculated by measuring the overall droplet diameter and the oil‐phase diameter, converting both diameters to volumes, and subtracting the oil‐phase volume from the total droplet volume to yield the interfacial volume.

### 4.4 Cell Culture and Cytotoxicity Assays

Human colorectal adenocarcinoma (Caco‐2) cells were maintained in MEM at 37 °C in a humidified atmosphere containing 5% CO_2_. Cytotoxicity of WPH and Pip was evaluated using the Cell Counting Kit‐8 (CCK‐8) assay. Caco‐2 cells were seeded into 96‐well plates and grown to confluence, then treated with diluted WPH or Pip for 24 h. After treatment, the medium was removed and 100 µL of diluted CCK‐8 reagent was added to each well. Plates were incubated for 1 h, and absorbance was measured at 450 nm using a microplate reader (SpectraMax iD3, molecular Devices, CA). Cell viability was calculated from the absorbance values.

### 4.5 WPH Transport Efficiency

In Transwell assays, 0.5 mL HBSS was added to the apical chamber and 1.5 mL HBSS to the basolateral chamber for equilibration. After 30 min, the buffers were removed. The apical chamber was then dosed with 0.5 mL of WPH solution (1 mg/mL) or 0.5 mL of a mixture of WPH (1 mg/mL) and Pip (10 µg/mL), while the control wells received 0.5 mL HBSS. The concentrations of WPH and Pip were selected based on CCK‐8 assay results, and the highest concentrations that maintained cell viability above 80% were chosen for use. Plates were incubated at 37 °C with 5% CO_2_ for 2 h, after which samples were collected from the basolateral chamber to determine WPH transport. WPH concentrations were quantified using the o‐phthalaldehyde (OPA) method by measuring absorbance at 340 nm with a microplate reader, with L‐serine as the calibration standard [50].

### 4.6 Pre‐Treatment of Caco‐2 Cell

10 µg/mL Pip was added to the apical chamber of Transwell inserts, and cells were incubated for 2 h under this condition, which was designated the “Pip‐treated” group. After 2 h of Pip exposure, Pip was removed and replaced with fresh MEM, and cells were incubated for an additional 18 h. This condition was designated the “Pip‐removed” group. For the “JNK inhibitor pretreated” group, Caco‐2 monolayers in the apical chamber were pretreated with the JNK‐specific inhibitor SP600125, then washed three times with PBS (0.01 M, pH 7.4) before exposure to 10 µg/mL Pip for 2 h. The apical chamber receiving MEM only served as the blank group.

### 4.7 Immunofluorescence Staining

After the respective pretreatments, cells were fixed with 4% paraformaldehyde for 20 min and permeabilized with 0.1% Triton X‐100 for 15 min. Samples were blocked with 5% goat serum for 2 h and then incubated with diluted anti‐ZO‐1 and anti‐Occludin overnight at 4 °C. After washing with PBS, fluorescently labeled secondary antibodies were applied for 2 h. Following final washes and mounting, images were acquired using a fluorescence microscope (DM4 B, Leica, Germany).

### 4.8 Quantitative Real‐Time PCR (RT‐qPCR)

Total RNA was extracted from pretreated cells using a column‐based kit according to the manufacturer's instructions. Complementary DNA (cDNA) was synthesized from the extracted RNA using an M‐MLV reverse transcription kit. RT–qPCR was performed on a StepOne Plus real‐time PCR system (Thermo Fisher Scientific, USA) with each sample run in triplicate. Primer sequences were listed in Table S3.

### 4.9 Western Blot

Culture medium was carefully removed and cells were washed three times with PBS. Cells were lysed in 100 µL RIPA buffer for 30 min, followed by sonication and centrifugation to collect the protein‐containing supernatant. Equal concentrations of protein samples were mixed with loading buffer and loaded onto SDS–PAGE gels prepared according to the manufacturer's instructions. After electrophoretic separation, proteins were transferred to PVDF membranes. Primary antibodies used for Western blotting included anti‐ZO‐1 (1:1000) and anti‐Occludin (1:1000), anti‐β‐actin (1:1000) was used as the loading control to check for protein loading. The secondary antibody was HRP‐conjugated goat anti‐rabbit IgG (1:2000). Bands were visualized using ECL chemiluminescence and quantified with ImageJ software (National Institutes of Health).

### 4.10 Changes in Relative Resistance

In Transwell inserts, 0.5 mL of MEM containing 10 µg/mL Pip was added to the apical chamber and 1.5 mL MEM to the basolateral chamber. After 2 h of exposure, chambers were washed with PBS and replaced with fresh MEM, followed by an additional 18 h incubation. Transepithelial electrical resistance (TEER) was measured periodically throughout the experiment, and relative changes in resistance were calculated using the blank (MEM‐only) group as the control.

### 4.11 Animal

Male Sprague Dawley (SD) rats (6–7 weeks old) and C57BL/6J mice (6–7 weeks old) were obtained from SPF (Beijing) Biotechnology Co., Ltd. All animal procedures were conducted in accordance with the Guide for the Care and Use of Laboratory Animals and were approved by the Institutional Animal Care and Use Committee of China Agricultural University (approval no. SYXK(Beijing) AW91904202‐4‐2). Animals had ad libitum access to sterilized water and standard chow throughout the study.

### 4.12 True Protein Digestibility

After a 7‐day acclimation period, 12 male SD rats were randomly assigned to four groups: WP, WP+Pip, WP(Pip), and Control. The WP group received WP by oral gavage doses at 3.15 g/kg body weight (bw). The WP+Pip group received WP (3.15 g/kg bw) and free Pip (0.63 mg/kg bw), whereas the WP(Pip) group received the WP(Pip) containing the same doses of WP (3.15 g/kg bw) and Pip (0.63 mg/kg bw). The Control group received PBS by oral gavage. During the dosing period, animals were provided a protein‐free diet and the amount of food intake was recorded. For determination of true protein digestibility, all feces were collected within 12 h after gavage, dried to constant weight, and weighed. Fecal nitrogen content was then measured by the Kjeldahl method. The true protein digestibility was calculated as follow:

$$\begin{array}{ccc} & & True\;protein\;digestibility\;\left(\%\right)=\\ & & \frac{\begin{array}{c} C_{intake\;nitrogen}-(C_{feces\;nitrogen\;in\;tested\;group}\\ \;-\;C_{feces\;nitrogen\;in\;control\;group}) \end{array}}{C_{intake\;nitrogen}} \end{array}$$

### 4.13 Serum Free Amino Acids

After a 7‐day acclimation period, 9 male SD rats were randomly assigned to three groups: WP, WP+Pip, and WP(Pip). The dosing regimens for WP, WP+Pip, and WP(Pip) were consistent with those described in Section 4.12. To quantify serum free amino acids, blood was collected at designated time points and centrifuged at 3000 rpm for 15 min to obtain serum. Serum free amino acid concentrations were measured using a commercial amino acid assay kit according to the manufacturer's instructions, and absorbance was read at 570 nm using a microplate reader.

### 4.14 Single‐Dose Pharmacokinetics of Pip

After a 7‐day acclimation period, 9 male SD rats were randomly assigned to 3 groups: Pip, WP+Pip, and WP(Pip) group. The Pip group received Pip by oral gavage at 20 mg/kg bw. The WP+Pip group received WP (3.15 g/kg bw) and free Pip (20 mg/kg bw), whereas the WP(Pip) group received the WP(Pip) with the same doses of WP (3.15 g/kg bw) and Pip (20 mg/kg bw). To ensure that plasma Pip concentrations exceeded the assay limit of detection, preliminary optimization experiments established a Pip dose at 20 mg/kg bw. Blood samples were collected at designated time points and centrifuged at 3,000 rpm for 15 min to obtain plasma. For plasma sample preparation, 100 µL of plasma was mixed with 300 µL methanol, vortexed thoroughly, and centrifuged at 12,000 rpm for 20 min at 4 °C. Pip concentrations in the plasma supernatants were determined by UPLC and quantified using an external standard calibration curve.

### 4.15 Fractional Synthesis Rate (FSR) of Skeletal Muscle Protein

FSR was determined by Flooding method [51]. 12 Male SD rats were randomized into four groups and fasted for 12 h prior to the experiment. The dosing regimens for WP, WP+Pip, and WP(Pip) were consistent with those described in Section 4.12. 45 min after gavage, the rats received an intravenous injection of L‐phenylalanine (RING‐D5) at 45 mg/kg bw via the tail vein. 15 min after injection, animals were euthanized and blood and skeletal muscle were collected.

Muscle samples were pulverized under liquid nitrogen, washed with HClO_4_, and centrifuged to collect the protein pellet. Protein‐bound L‐phenylalanine (RING‐D5) was released by hydrolyzing the pellet in 6 M HCl at 110 °C for 24 h. Serum proteins were precipitated with 7.5% (v/v) trichloroacetic acid, and the resulting supernatants were passed through a 0.22 µm filter for analysis of free L‐phenylalanine (RING‐D5). Quantitative analysis of L‐phenylalanine (RING‐D5) in muscle hydrolysates and serum fractions was performed by Triple Quad LC/MS/MS (1200‐6460, Agilent, St. Clara, CA, U.S.), using L‐phenylalanine (RING‐D5) standards for external calibration. The elution gradient was detailed in Table S4.

### 4.16 Western Blot

0.10 g of rat hindlimb muscle tissue was homogenized in 1 mL RIPA lysis buffer, and the homogenates were centrifuged using a microcentrifuge to collect the supernatant. The subsequent procedures followed the protocol described in Section 4.9. Primary antibodies used for analysis included anti‐mTOR (1:1000), anti‐S6K1 (1:1000), and anti‐4E‐BP1 (1:1000). The secondary antibody was HRP‐conjugated goat anti‐rabbit IgG (1:2000).

### 4.17 Animals Care

For healthy mice, 30 male C57BL/6J mice were acclimated for 7 days and then randomly divided into four groups (12 in control group and 6 in other groups, respectively). The WP group received WP by oral gavage doses at 1 g/kg body weight (bw). The WP+Pip group received WP (1 g/kg bw) and free Pip (0.91 mg/kg bw), whereas the WP(Pip) group received the WP(Pip) containing the same doses at WP (1 g/kg bw) and Pip (0.91 mg/kg bw). The control groups included Con‐SED (control group remained at rest, without a standardized exercise) and Con‐EXE (control group with a standardized exercise), both of which received an equal volume of PBS by oral gavage. Notably, Con‐SED and Con‐EXE received identical treatments before day 36. Oral gavage was performed daily at the same time in the morning for 35 consecutive days. From day 29 onward, body composition analysis by NMR, forelimb grip strength test, and exhaustive swimming test were conducted. On day 36, mice were subjected to a 15‐min forced swimming test without load, after which they were immediately sacrificed to collect blood and gastrocnemius samples, which were stored at −80 °C until further analysis. The gastrocnemius was fixed in 4% paraformaldehyde, embedded in paraffin, sectioned, and subjected to hematoxylin–eosin (H&E) staining for histological evaluation.

For the Dex‐induced sarcopenia model, 25 male C57BL/6J mice were acclimated for 7 days and then randomly assigned to five groups. All mice except the control group received daily intraperitoneal injections of Dex (20 mg/kg) to induce sarcopenia. To achieve therapeutic intervention, the WP group received WP by oral gavage doses at 1 g/kg body weight (bw). The WP+Pip group received WP (1 g/kg bw) and free Pip (0.91 mg/kg bw), whereas the WP(Pip) group received the WP(Pip) containing the same doses at WP (1 g/kg bw) and Pip (0.91 mg/kg bw). Both the control and Dex groups received an equal volume of PBS solution by oral gavage. Oral gavage was performed once daily at the same time in the morning for 14 consecutive days. At the end of the intervention, body composition analysis by NMR and forelimb grip strength test were tested and mice were sacrificed. Blood and skeletal muscle samples were collected. Serum was stored at −80 °C for subsequent analyses. Fresh muscle tissues were weighed. The Gastrocnemius was fixed in 4% paraformaldehyde, embedded in paraffin, sectioned, and subjected to hematoxylin–eosin (H&E) staining for histological evaluation.

### 4.18 Phenotypic Assays

For body composition analysis by NMR, lean mass and fat mass of live mice were directly measured using a body composition analyzer. For forelimb grip strength testing, a grip strength meter for rodents was used, and each mouse was tested in triplicate to obtain parallel measurements. For the swimming‐to‐exhaustion test, mice were fasted for 12 h prior to the experiment and orally gavaged with PBS, WP, WP+Pip, or WP(Pip) 30 min before testing. A lead weight corresponding to 5% of body weight was securely attached to the base of the tail. During the test, necessary manual intervention was applied to ensure continuous swimming activity. Exhaustion was defined as the point when the mouse's head remained submerged under water for more than 5 s. Immediately after exhaustion, mice were rescued and rewarmed to restore normal body temperature.

### 4.19 Serum Biochemical Parameters Analysis

Serum Lactate, creatine kinase (CK), lactate dehydrogenase (LDH), superoxide dismutase (SOD), malondialdehyde (MDA), and glutathione peroxidase (GSH‐Px) were determined using commercial assay kits (Nanjing Jiancheng Bioengineering Institute, Jiangsu, China). Serum LPS, IL‐1β, MyoD and MyoG concentrations were measured using enzyme‐linked immunosorbent assay (ELISA) kits (Beijing Bo Rui Chang Yuan Technology Co., Ltd., Beijing, China).

### 4.20 Statistical Analysis

Unless otherwise noted, all experiments were performed with at least three replicates. Data were presented as mean ± standard deviation. Differences between two groups were assessed using Student's t‐test, and comparisons among multiple groups were performed by one‐way analysis of variance (ANOVA). P values < 0.05 were considered statistically significant.

## Author Contributions

**Q.Y**. and **K.W**. contributed equally to this work. **Q.Y**. curated data, performed formal analysis and visualization, worked with the software, and developed methodology. **K.W**. performed formal analysis and visualization, developed methodology, and wrote the original draft. **B.W**. curated data and developed the methodology. **G.S**. developed the methodology and worked with the software. **C.S**. acquired resources. **X.L**. developed the methodology and worked with the software. **X.L**. conceptualized the study. **Z.M**. developed methodology. **M.L**. conceptualized the study. **J.L**. edited the final draft. **Y.L**. conceptualized the study, acquired funds and resources, performed supervision, and wrote, reviewed, and edited the final draft. All the authors discussed the results and commented on the manuscript.

## Conflicts of Interest

The authors declare no conflict of interest.

## Supporting information




**Supporting File**: advs74993‐sup‐0001‐SuppMat.docx.

## Data Availability

The data that support the findings of this study are available from the corresponding author upon reasonable request.;
