# Supplementary material for: The Enhanced Whey Protein Oral Bioavailability and Muscle Anabolism Ability by a Simple and Effective Piperine‐Whey Protein Synergistic Codelivery System
Source: Adv Sci (Weinh). 2026 Mar 26;13(32):e22455. doi: 10.1002/advs.202522455 (PMC13252643; doi:10.1002/advs.202522455)
Supplement: Supplementary file 1 — Supporting File: advs74993‐sup‐0001‐SuppMat.docx. [file ADVS-13-e22455-s001.docx]

**Supporting information**

**The enhanced whey protein oral bioavailability and muscle anabolism ability by a simple and effective piperine-whey protein synergistic codelivery system**

*Qing Yue^1,#^，Kaiwen Wu^1,#^, Bing Wei^2^, Guiling Song^2^, Congyu Sun^2^, Xing Li^1^, Xiangyu Liu^1^,Zhaoxiang Ma^1^, Mingchun Lv^3^, Jasper Landman^4^, Yuan Li^1,^**

*^1^ Q. Yue, K. Wu, X. Li, X. Liu, Z. Ma, Y. Li,*

*Research Center of Food Colloids and Delivery of Functionality, College of Food Science and Nutritional Engineering, China Agricultural University, Beijing 100083, China*

*Email: yuanli@cau.edu.cn*

*^2^ B. Wei, G. Song, C. Sun*

*Beijing Competitor Sports Science Technology Joint Stock Co. Ltd, Beijing 102299, China*

*^3^ M. Lv*

*Frontier Technology Research Institute of China Agricultural University in Shenzhen, 518119, China*

^4^ *J. Landman*

*Physics and Physical Chemistry of Foods, Wageningen University and Research, Bornse Weilanden 9, Wageningen, 6708 WG, The Netherlands*

# Q. Yue and K. Wu contributed equally to this work.

*Corresponding author Prof. Y. Li, E-mail: yuanli@cau.edu.cn

**Table S1.** Pharmacokinetic parameters of Pip *in vivo*.

| Sample | C_max_ (μg/mL) | T_max_ (min) | AUC_0-24 h_ (μg×h/mL) |
| --- | --- | --- | --- |
| Pip | 0.21±0.06 | 30 | 1.27±0.29 |
| WP+Pip | 1.11±0.17 | 60 | 6.47±0.45 |
| WP(Pip) | 2.58±0.44 | 15 | 11.30±0.83 |

**Table S2.** SGF and SIF composition.

|  | SGF mg /30 mL | SIF mg / 30 mL |
| --- | --- | --- |
| NaCl | 82.8 | 67.3 |
| KH2PO4 | 3.7 | 3.3 |
| NaHCO3 | 63 | 214.2 |
| Enzyme | 40 mg Pepsin | 24 mg Trypsin |
| Water | Up to 30 mL | Up to 30 mL |
| pH | 2.0 | 7.0 |

**Table S3.** Sequences of the primers used for RT-qPCR.

| Gene | Forward Sequences (5’→3’) | Reverse Sequences (5’→3’) |
| --- | --- | --- |
| ZO-1 | CCATTGTGATAGCTCATCGTTT | CACTTGTTTTGCCAGGTTTTA |
| Occludin | CCAATGTCGAGGAGTGGG | CGCTGCTGTAACGAGGCT |
| β-Actin | GCACAGTCAAGGCCGAGAAT | GCCTTCTCCATGGTGGTGAA |

**Table S4.** Gradient elution procedure.

| Time (min) | Mobile phase A (%) | Mobile phase B (%) |
| --- | --- | --- |
| 0 | 100 | 0 |
| 4 | 85 | 15 |
| 10 | 0 | 100 |
| 13 | 100 | 0 |
| 16 | 100 | 0 |


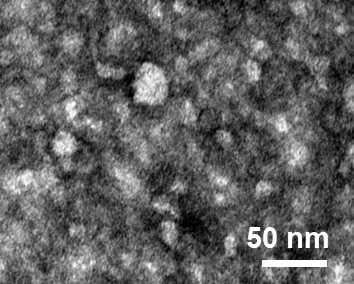


**Figure S1.** TEM image of WP (10 wt.%). Scale bar = 50 nm.


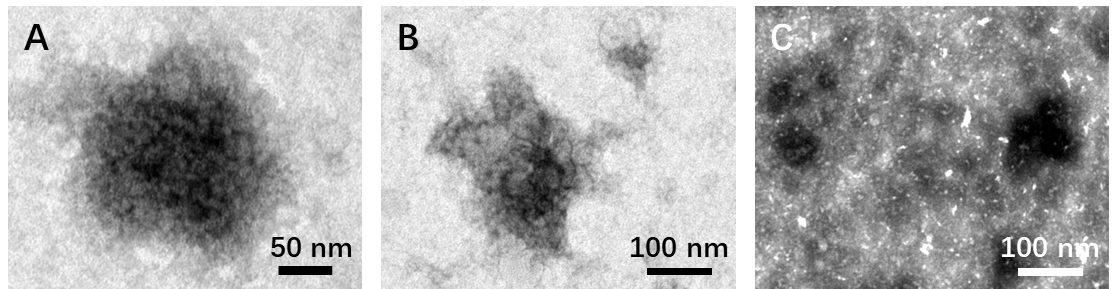


**Figure S2.** TEM image of simulated digestion of WP+Pip. (A) WP+Pip in DI water. The scale bar = 50 nm. (B) WP+Pip in SGF for 60 min. The scale bar = 100 nm. (C) WP+Pip in SIF for 60 min. The scale bar = 100 nm.


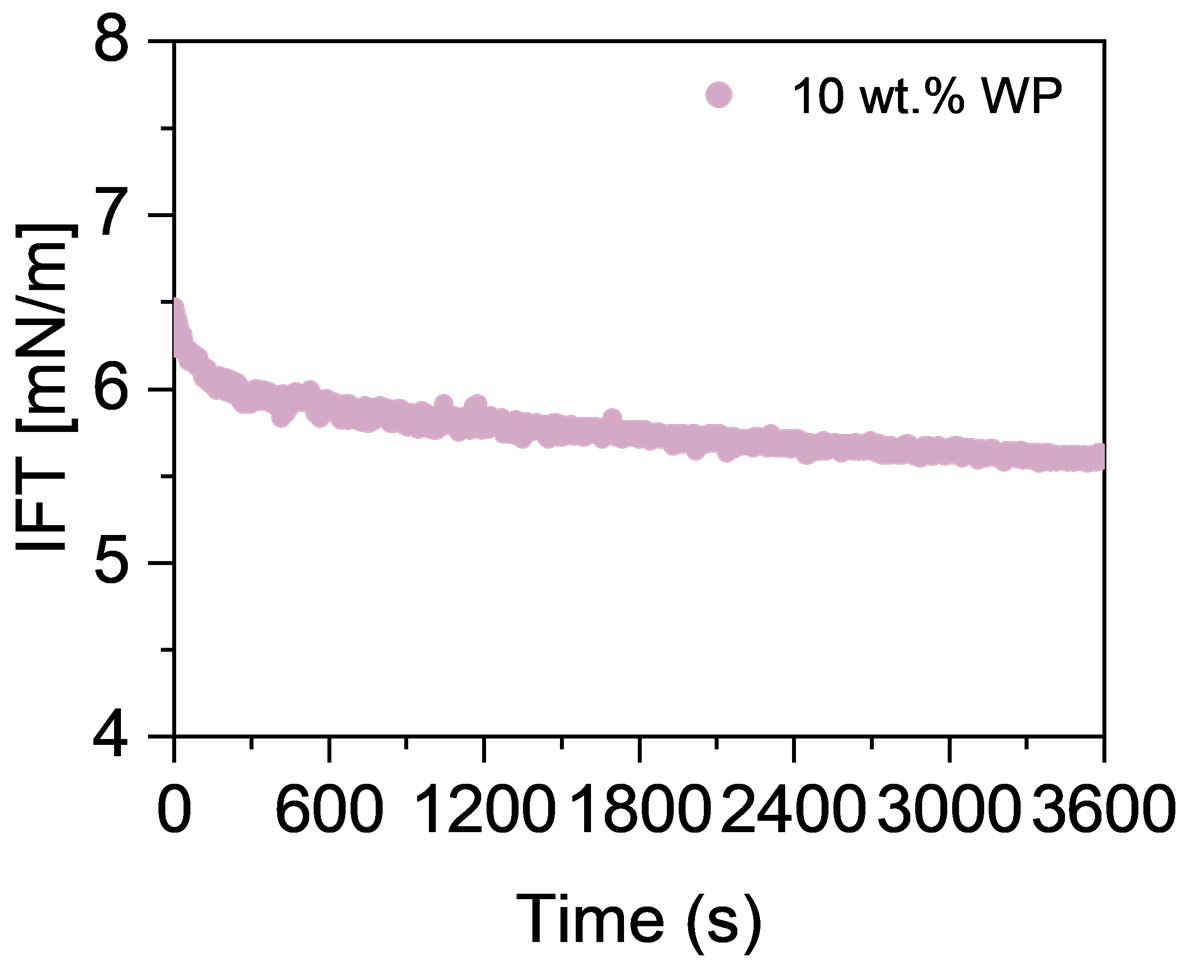


**Figure S3.** Interfacial tension of 10 wt.% WP in 3600 s.


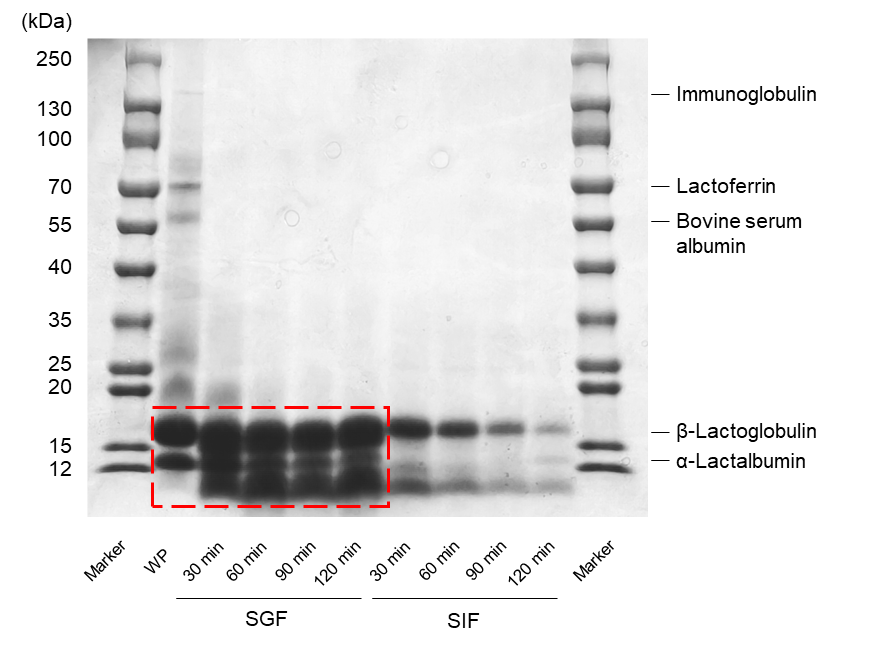


**Figure S4.** SDS-PAGE profile of the gastrointestinal digestion of whey protein. The main components of WP, β-lactoglobulin and α-lactalbumin, are stable during the SGF stage.


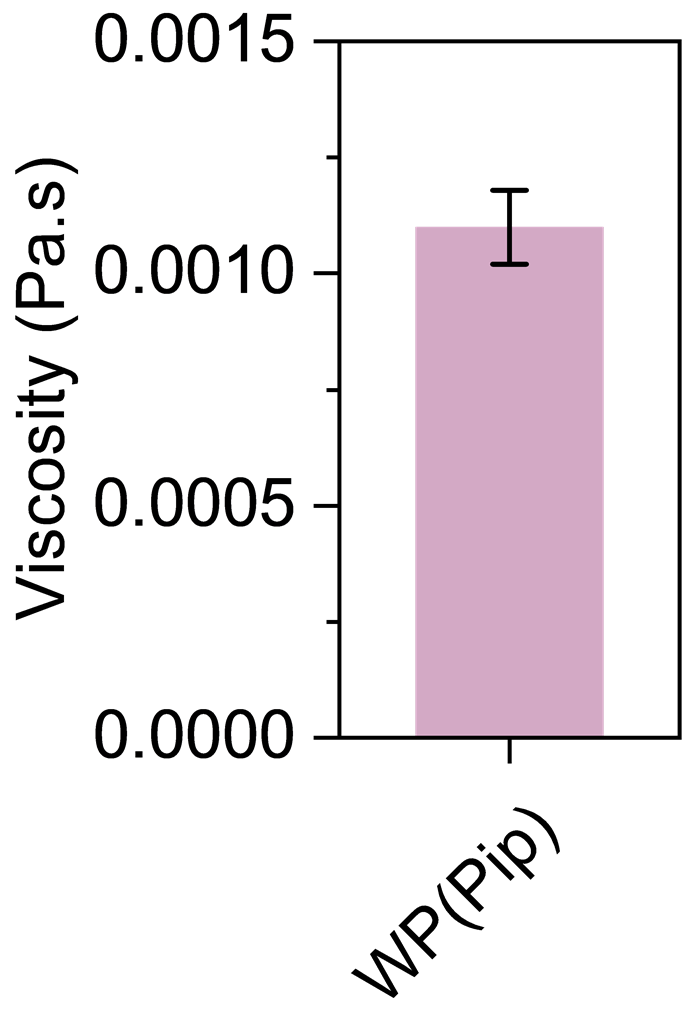


**Figure S5.** Viscosity of WP(Pip).


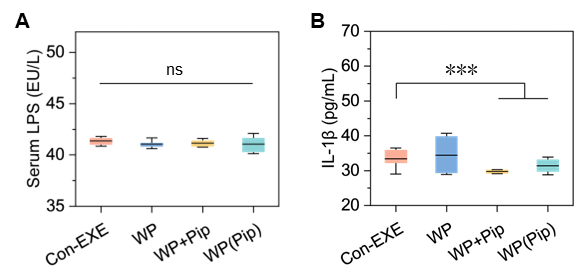


**Figure S6.** WP+Pip and WP(Pip) did not cause leakage of harmful bacteria or metabolites following reversible TJ regulation. (A) Serum LPS. (B) IL-1β in mice supplemented with PBS, WP, WP+Pip, or WP(Pip) for 36 days. Equivalent daily doses of WP were administered in the WP, WP+Pip, and WP(Pip) groups. Data are presented as mean ± SD (n = 6). Statistical significance was assessed using Student’s t-test. “ns” indicates not significant, and ***p ≤ 0.001.


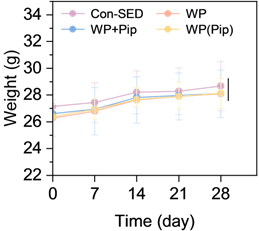


**Figure S7.** Body weight changes of healthy mice after oral administration of different samples for 35 days. Data are presented as mean ± SD (n = 6). Statistical significance was assessed by Student’s t-test. “ns” means not significant. *p ≤ 0.05, **p ≤ 0.01, ***p ≤ 0.001.


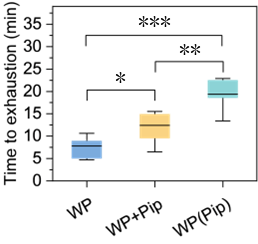


**Figure S8.** Time to exhaustion of healthy mice after 28 days of supplementation with different WP formulations. Data are presented as mean ± SD (n = 6). Statistical significance was assessed by Student’s t-test. *p ≤ 0.05, **p ≤ 0.01, ***p ≤ 0.001.


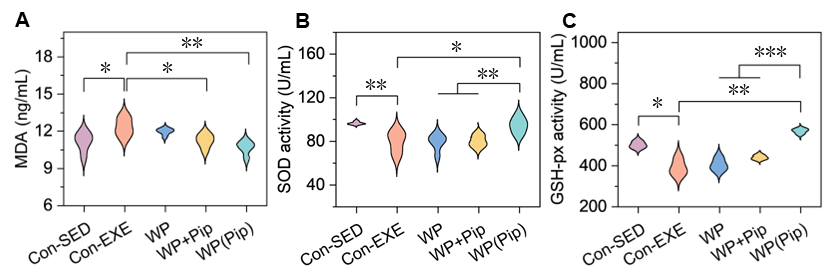


**Figure S9.** WP(Pip) relieved post-exercise oxidative stress by enhancing endogenous antioxidant enzyme activity. (A) Serum MDA content. (B) Serum SOD activity. (C) Serum GSH-px activity in mice supplemented with PBS, WP, WP+Pip, or WP(Pip) for 36 days. Equivalent daily doses of WP were administered in the WP, WP+Pip, and WP(Pip) groups. Data are presented as mean ± SD (n = 6). Statistical significance was assessed by Student’s t-test. *p ≤ 0.05, **p ≤ 0.01, ***p ≤ 0.001.


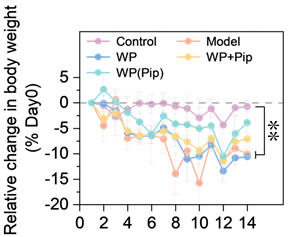


**Figure S10.** Body weight change rate of different intervention groups after intraperitoneal injection of Dex for 14 days. Data are presented as mean ± SD (n = 5). Statistical significance was assessed by Student’s t-test. **p ≤ 0.01.
